# Research trends of acupuncture therapy on insomnia in two decades (from 1999 to 2018):a bibliometric analysis

**DOI:** 10.1186/s12906-019-2606-5

**Published:** 2019-08-22

**Authors:** Wenya Pei, Rui Peng, Yuan Gu, Xiaohong Zhou, Jingwen Ruan

**Affiliations:** 1 Department of acupuncture, The First Affiliated Hospital, Sun Yat-sen University, Guangzhou, Guangdong China; 2 College of Acupuncture and Orthopedics, Hubei University of Chinese Medicine, Wuhan, Hubei China; 3 Guangdong Provincial Hospital of Traditional Chinese Medicine, Guangzhou, Guangdong China

**Keywords:** Acupuncture, Insomnia, Bibliometric analysis

## Abstract

**Objectives:**

We aimed to evaluate the global scientific output of research of acupuncture on insomnia and explore the hotspots and frontiers from 1999 to 2018, by using bibliometric methods.

**Methods:**

Articles about acupuncture therapy on insomnia were retrieved from the Web of Science Core Collection (WoSCC). We used Citespace V to analyze publication years, journals, countries, institutions, authors and cited authors. We plotted the reference co-citation network and key words to analyze the research hotspots and trends.

**Results:**

Until August 31, 2018 31, 2018, a total of 292 records in acupuncture therapy on insomnia research were identified from 1999 to 2018. The number and rate of the annual publication gradually increased. Respectively, SLEEP and J NEUROPSYCH CLIN N (journal of neuropsychiatry and clinical neurosciences) ranked the first in the frequency and centality of cited joural. Among countries, China ranked highest in the number of publications and the top 3 institutes were in Hong Kong. Chung KF and Yeung WF were the most productive authors and YEUNG WF ranked the first in the cited authors. In the ranking of frequency and in cited reference, the first was the article published in by CAO HJ and KALAVAPALLI R. ‘Randomized controlled trial’ had a high frequncy and centrality in keyword.

**Conclusions:**

A higher degree of acceptance acupuncture was obtained in the Asian. Recently, systematic reviews and clinic trials most focused on electrocacupuncture and acupressure among the acupuncture therapy.

## Introduction

Insomnia is a common symptom or disorder which means patients suffer difficulties in initiating or maintaining sleep or impaired daytime functioning accompanying with early morning awakening [1]. Approximately 10 to 20% of the population wordwide have problem in sleep quality and the prevalence of insomnia is 33 to 50% in adults [2, 3]. Insomnia could cause burdens to individuals and society by increasing risk of psychological disorders such as anxiety, depression, immune functioning, cardiovascular disease and even suicide [4–7]. Sleep-wake regulation play an important role in genetic, molecular mechanism, cellular mechanism of sleep and insomnia [8]. In present, the main treatment is recommended for insomnia are cognitive-behavioral therapy and benzodiazepine receptor, which are focused on sleep quality and quantity, daytime function and sleep latency [9]. But the short-term effect, adverse events, rebound insomnia, development of tolerance and other risk still exist [10].

Gradually, the usage and acceptance of Complementary and Alternative Medicine (CAM) is increasing in the wordwide, it has become an option for insomnia [11–14]. As one of complementary treatment modalities, previous studies have shown that acupuncture has hypnotic effects, especially in primary insomnia, depression-related insomnia, and cancer-related insomnia [15–17]. Through regulating neurotransmitters and hormonal factors, acupuncture could modulate sleep and wakefulness to improve the quality of sleep [18, 19].

Bibliometric analysis which combines mathematical and statistical methods could generating and organizing knowledge structure and development through information processing [20, 21]. Citespace V is a the bibliometric visualization tools which is widely used to visualize and analyze emerging trends and transition patterns in scientific literature [22–24]. To gain insights into research trend and hot spots in the field of acupuncture therapy on insomnia, we performed a bibliometric analysis of articles from 1998 to 2018 by using CiteSpace V.

## Metarials & methods

### Data collection

The data of this review was collected from the Web of Science (WoS) including SCI-EXPANDED, SSCI, A&HCI, CPCI-S, CPCI-SSH, BKCI-S, BKCI-SSH, ESCI, and CCR-EXPANDE on August 31,2018. The search strategy consisted of three parts. First, we listed the index words about ‘acupuncture therapy’ such as acupuncture, acupuncture point, ear acupuncture, body acupuncture, auricular acupuncture, electroacupuncture, electro-acupuncture, moxi-bustion. All language and all document type were included with the timespan from 1999 to 2018. By this query, 17604 records was generated.

Second, the topic search focused on the index words about ‘insomnia’ such as sleep initiation, maintenance disorders, disorders of initiating and maintaining sleep, primary insomnia, transient insomnia, secondary insomnia, sleeplessness, insomnia disorder, sleep initiation dysfunction. The language, the document type and the timespan were setted as same as the first query. This query resulted in 21893 records.

Then, we combined the first query and the second query to find documents focused on the acupuncture in insomnia. A total of 292 records was obtained. The topic search queries were in Table 1.

**Table 1:** The topic search queries

Set	Results	Search query
#3	292	#2 AND #1 Indexes = SCI-EXPANDED,SSCI,A&HCI,CPCI-S,CPCI-SSH,BKCI-S,BKCI-SSH,ESCI,CCR-EXPANDE Timespan = 1999–2018
#2	21893	(TS = (insomnia OR sleep Initiation and maintenance disorders OR disorders of initiating and maintaining sleep OR primary insomnia OR transient insomnia OR secondary insomnia OR sleeplessness OR insomnia disorder OR sleep initiation dysfunction)) Indexes = SCI-EXPANDED,SSCI,A&HCI,CPCI-S,CPCI-SSH,BKCI-S,BKCI-SSH,ESCI,CCR-EXPANDE Timespan = 1999–2018
#1	17604	(TS = (acupuncture therapy OR acupuncture OR acupuncture point OR Acupuncture, Ear OR body acupuncture OR Auricular Acupuncture OR Electroacupuncture OR electro-acupuncture OR Moxibustion)) Indexes = SCI-EXPANDED,SSCI,A&HCI,CPCI-S,CPCI-SSH,BKCI-S,BKCI-SSH,ESCI,CCR-EXPANDE Timespan = 1999–2018

### Analysis tool

Citespace V is a bibliometric analysis visualization software based on the Java platform which for visualizing and analyzing network [22]. Citespace is mainly used to help analyze knowledge inflection points, research hotspots, evolution paths, knowledge structures and new trends in the knowledge field [25]. It focuses on simultaneously identifying the time, frequencies, and centralities of the co-occurrence networks [26]. Several types of bibliometric studies such as co-word analysis, author co-citation analysis, document co-citation analysis, and text and geospatial visualizations are supported by this software [27].

After importing data through Citespace, we could analysis the association between journals, explore collaboration networks between authors/institutes/countries, identify co-cited authors/references, capture keywords with strong citation bursts, and construct visualization maps of all items [28]. Nodes and links are used to make up of visualization knowledge maps. In a network, the betweenness centrality of a node which is the common form of structural metric is used to identify cluster members. The color of treerings represents the number of citations, warm colors mean the a citation burst, cold colors mean the opposite. Nodes with high centrality are usually regarded as turning points or pivotal points in a field.

## Results and discussion

### Annual publication

**Fig. 1:**
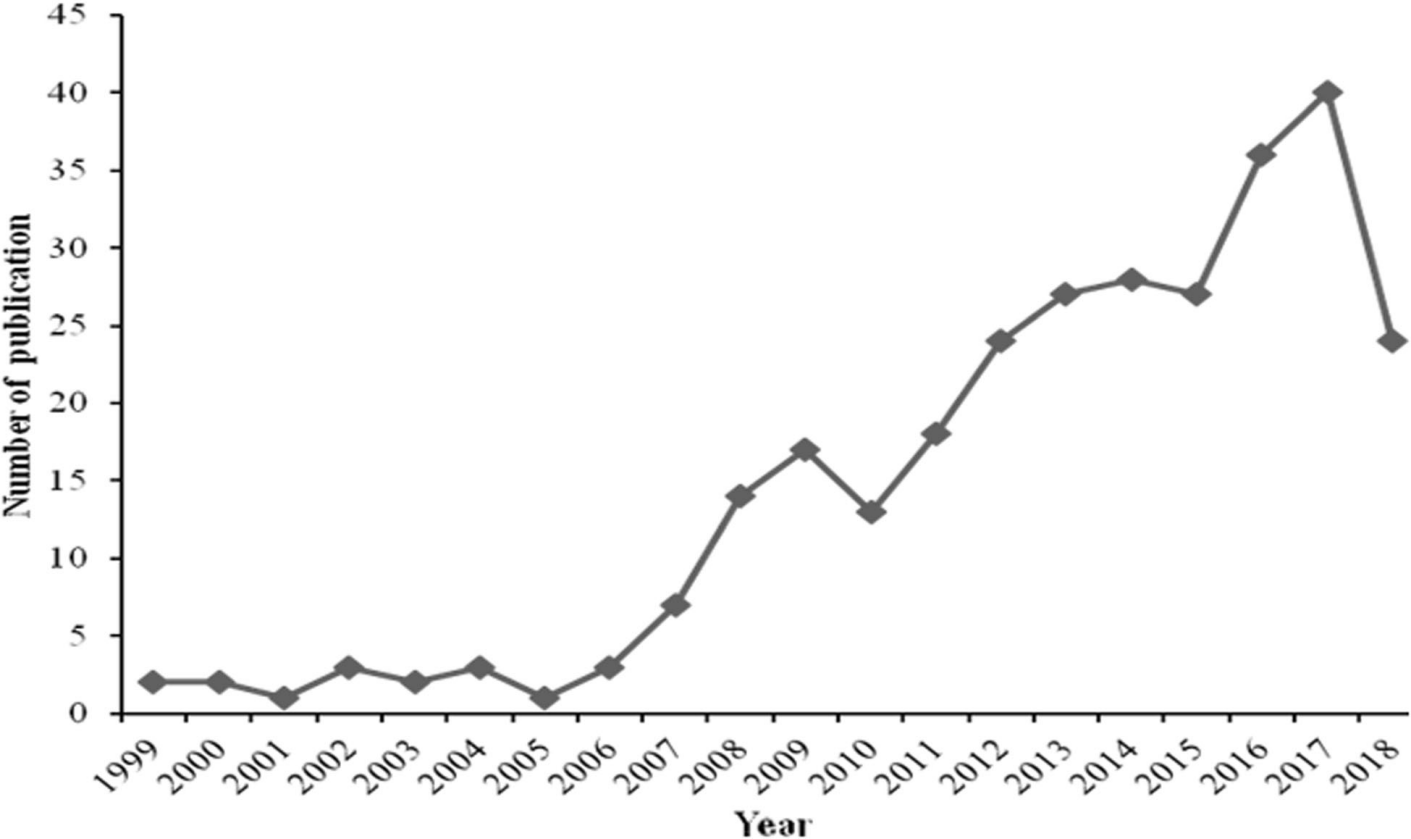
The annual number of publication on acupuncture in insomnia

In total, 292 records were included, the number of publications by year was presented in Fig. 1. Through the figure, we can find that the several stages in the research trends. In the first stage from 1999 to 2009, the number of publication rised from 2 references to 17 references in 2009. But the number decreased in 2010 and then the number restored to 18 in 2011. From 2010 to 2015, it was a stage which slowly increased. Although the number slight declined in 2015, but the recovery appeared quickly in 2016. The third stage was from 2015 to 2017 which the number of publication increased rapidly. The number in 2018 can’t reflected the publication of the whole year. From the trend of the number, we can see that more research is being carried out on the acupuncture therapy in insomnia.

### Analysis cited journal

A cited journal map was generated by a total of 8268 references through Citespace. SLEEP ranked the first in the frequency of cited joural, and J NEUROPSYCH CLIN N (journal of neuropsychiatry and clinical neurosciences) ranked the first in the centality (Table 2). It was interesting that J ALTERN COMPLEM MED (journal of alternative and complementry medicine) ranked both the second in the frequency and centality.

**Table 2:** Top 5 cited Journal related to acupuncture for insomnia

Rank	Cited Journal	Freq	Rank	Cited Journal	Centality
1	SLEEP	125	1	J NEUROPSYCH CLIN N	0.44
2	J ALTERN COMPLEM MED	106	2	J ALTERN COMPLEM MED	0.42
3	SLEEP MED	104	3	SLEEP	0.23
4	EVID-BASED COMPLALT	88	4	JAMA-J AM MED ASSOC	0.21
5	SLEEP MED REV	66	5	LANCET	0.13

**Fig. 2:**
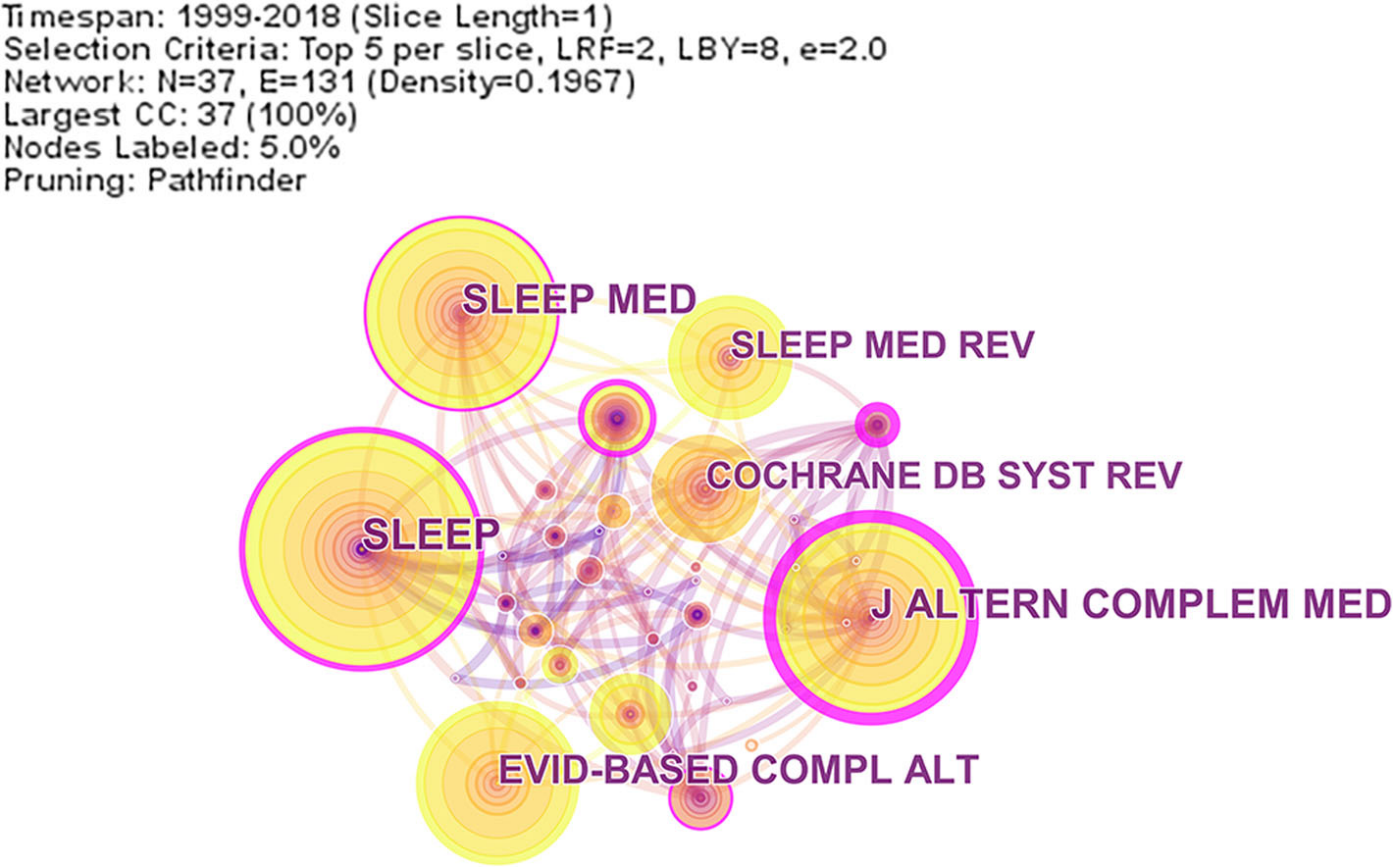
cited journal map of acupuncture for insomnia

Through the map in Fig. 2, the color of EVID-BASED COMPLALT (evidence-based complementary and alternative medicine) was red which meaned there was a cited/frequency burst. In the journal cited in 125 records of SLEEP, one article which focused on the systematic review of insomnia and complementary medicine got the largest citations [29]. In this article,evidences were provided to suppot the treatment of acupuncture therapy for chronic insomnia.

### Distribution of countries and institutes

One hundred and twenty records in a total of 292 records were published in China, which is the origin of acupuncture therapy. As the second country in the ranking list (Table 3), USA got 70 records which meaned the widely use of acupuncture in the treatment of insomnia in this country. And in Taiwan, England and South Korea, acupuncture therapy still attracted attention in the treatment of nisomnia. From the citations, more and more randomized and controlled trials were conducted to support the efficiency of acupuncture therapy in insomnia [30, 31]_._

**Table 3:** Top 5 country/region and institute related toacupuncture for insomnia

Rank	Country/Region	Freq	Institute	Freq
1	PEOPLES R CHINA	120	Univ Hong Kong	29
2	USA	70	Hong Kong Polytech Univ	13
3	TAIWAN	20	Hong Kong Baptist Univ	8
4	ENGLAND	12	Guangzhou Univ Chinese Med	8
5	SOUTH KOREA	9	China Med Univ	7

In 26 institutes which paied close attention in the field of acupuncture therapy in insomnia, the top 3 institutes were in Hong Kong (Table 3). They were University of Hong Kong, the Hong Kong Polytechnic University and Hong Kong Baptist University. Besides, Guangzhou University of Chinese Medcine and China Medcine University also interested in this field. In the aspect of evidence-based medicine, a few of systematic reviews were perfomed to provide evidences that acupressure, reflexology, and auricular acupressure could be beneficial for insomnia [14, 32].

### Analysis of author and cited author

**Fig. 3:**
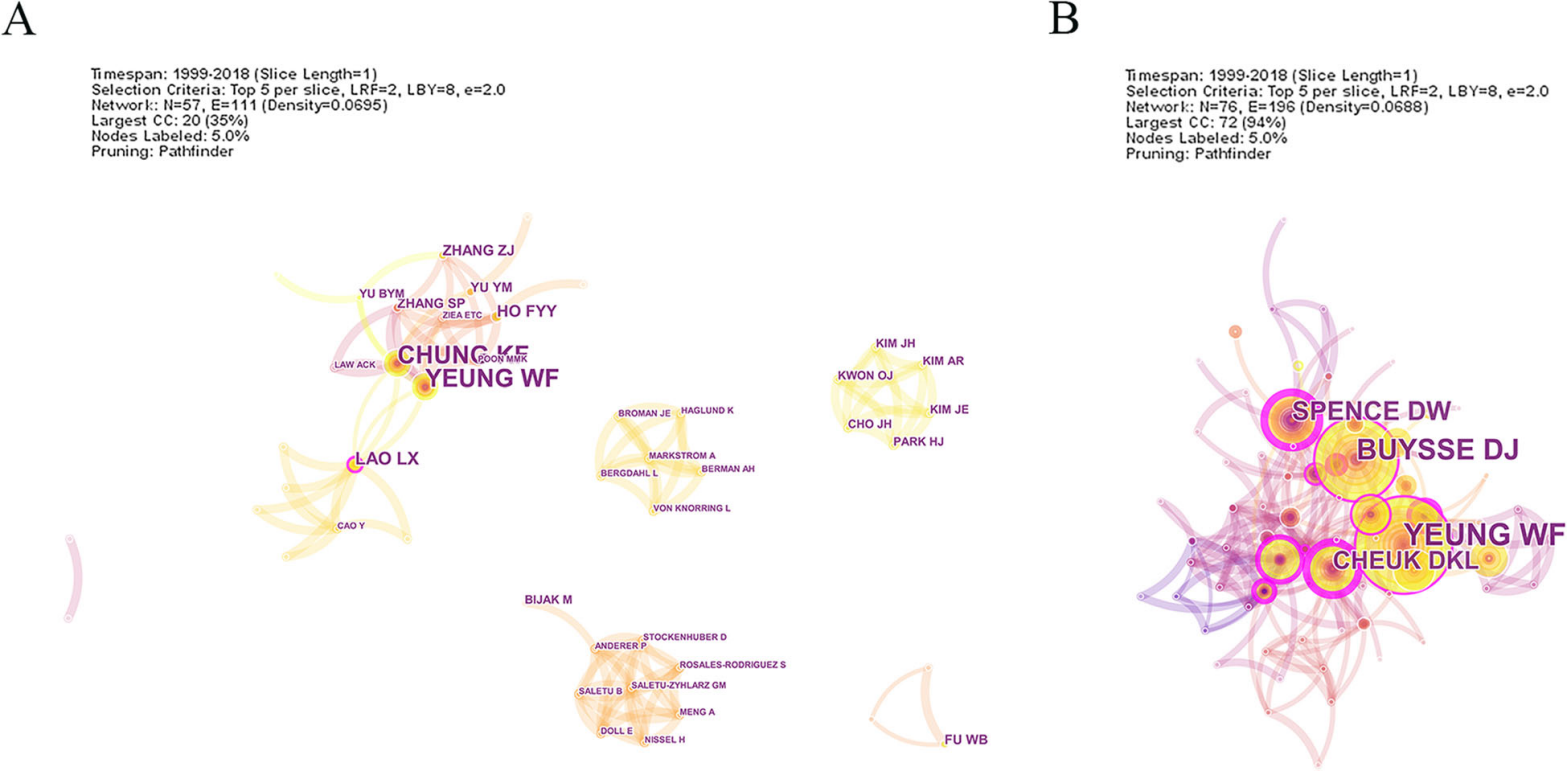
Author and cited-author map of acupuncture for insomnia. **a** The network map of author related to acupuncture for insomnia. **b** The network map of cited author related to acupuncture for insomnia

In the number of publication, Chung KF and Yeung WF were the most productive authors. They have cooprated with each other in the field focued on clinical observation and systematic evaluation [33, 34]. Liao LX, Ho FYY, Zhang ZJ also were active in this field. From the network map (Fig. 3), we could find closely notes among the top 5 authors and this identify the close cooperation in these professional authors. Most of them came from the University of Hong Kong and a research team has been established to specialize in the field of acupuncture therapy for insomnia.

Among 76 notes and 196 links, YEUNG WF ranked the first in the cited authors, followed by BUYSSE DJ, SPENCE DW, CHEUK DKL and MORIN CM (Table 4). From the summary of cluster, authors devoted their mind to systematic review, clinic trial and auricular acupuncture treatment which ranked top 3 in the keywords. BUYSSE DJ who was an specialist in sleep focused on the diagnose and treatment of insomnia [35]. SPENCE DW which ranked the third in the frequency but got the first centrality. A prepost clinical trial study was conducted by his team to identify the effective of acupuncture treatment to anxious patients with insomnia [18].

**Table 4:** Top 5 author and cited author related toacupuncture for insomnia

Rank	Author	Freq	Centrality	Cited author	Freq	Centrality
1	Chung KF	20	0.07	YEUNG WF	67	0.09
2	Yeung WF	20	0.07	BUYSSE DJ	56	0.09
3	Lao LX	8	0.11	SPENCE DW	34	0.57
4	Ho FYY	6	0.02	CHEUK DKL	33	0.27
5	Zhang ZJ	5	0	MORIN CM	32	0.26

### Analysis of cited reference

A total of 8268 references were generated from 292 records to analysis cited references. Setted with a timespan from 1999 to 2018 and a time slice of 1, top 5 most cited or occurred items from each slice were choosen to form the network map of cited references. With a modularity Q of 0.7258 and a means silhouette of 0.5163, the map consisted of 122 nodes and 285 links. The value of modularity Q and means silhouette meaned the clusters were rational.

According to the ranking of frequency in cited reference (Table 5), the first was the article published in 2009 by CAO HJ [36]. The article conducted a meta-analysis to confirm the benifical effect of acupuncture therapy and made a suggestion that large and rigorous designed trials were wanted. In the ranking list, three article were published by YEUNG WF respectively in SLEEP and SLEEP MEDICINE. One article was a systematic review the other two were about randomized controlled trials of electrocacupuncture.

**Table 5:** Top 5 cited reference related to acupuncture for insomnia

Rank	Cited Reference	Freq	Rank	Cited Reference	Centality
1	CAO HJ,2009	33	1	KALAVAPALLI R,2007	0.35
2	YEUNG WF,2009	27	2	YEUNG WF,2009	0.26
3	SPENCE DW,2004	22	3	TSAY SL,2004	0.2
4	YEUNG WF,2009	19	4	CHEN HY,2007	0.14
5	YEUNG WF,2011	18	5	CAO HJ,2009	0.13

Abbreviation: *Freq* frequency

In residual insomnia associated with major depressive disorder and primary insomnia, slight advantages were found of electroacupuncture compared with placebo acupuncture [31, 37]. The open prepost clinical trial study published by SPENCE DW which ranked the third reference made a breakthrough that acupuncture therapy may increase nocturnal melatonin secretion [18].

When ranked by betweenness centrality (Table 5), the first was a article published by KALAVAPALLI R which performed a systematic review with a conclusion that acupuncture therapy may be effective associated with other psychiatric or medical conditions [38]. TSAY SL conducted a randomized controlled trial to identify the effectiveness of acupressure and Transcutaneous Electrical Acupoint Stimulation (TEAS) on fatigue, sleep quality and depression in patients [39]. And the other paper published by CHEN HY focused on the efficacy and safety of auricular acupuncture treatment for insomnia through meta analysis [32]. And the annalysis found that Shenmen was the most commonly used auricular acupoints, followed by Heart, Occiput, Subcortex, Brain and Kidney.

**Fig. 4:**
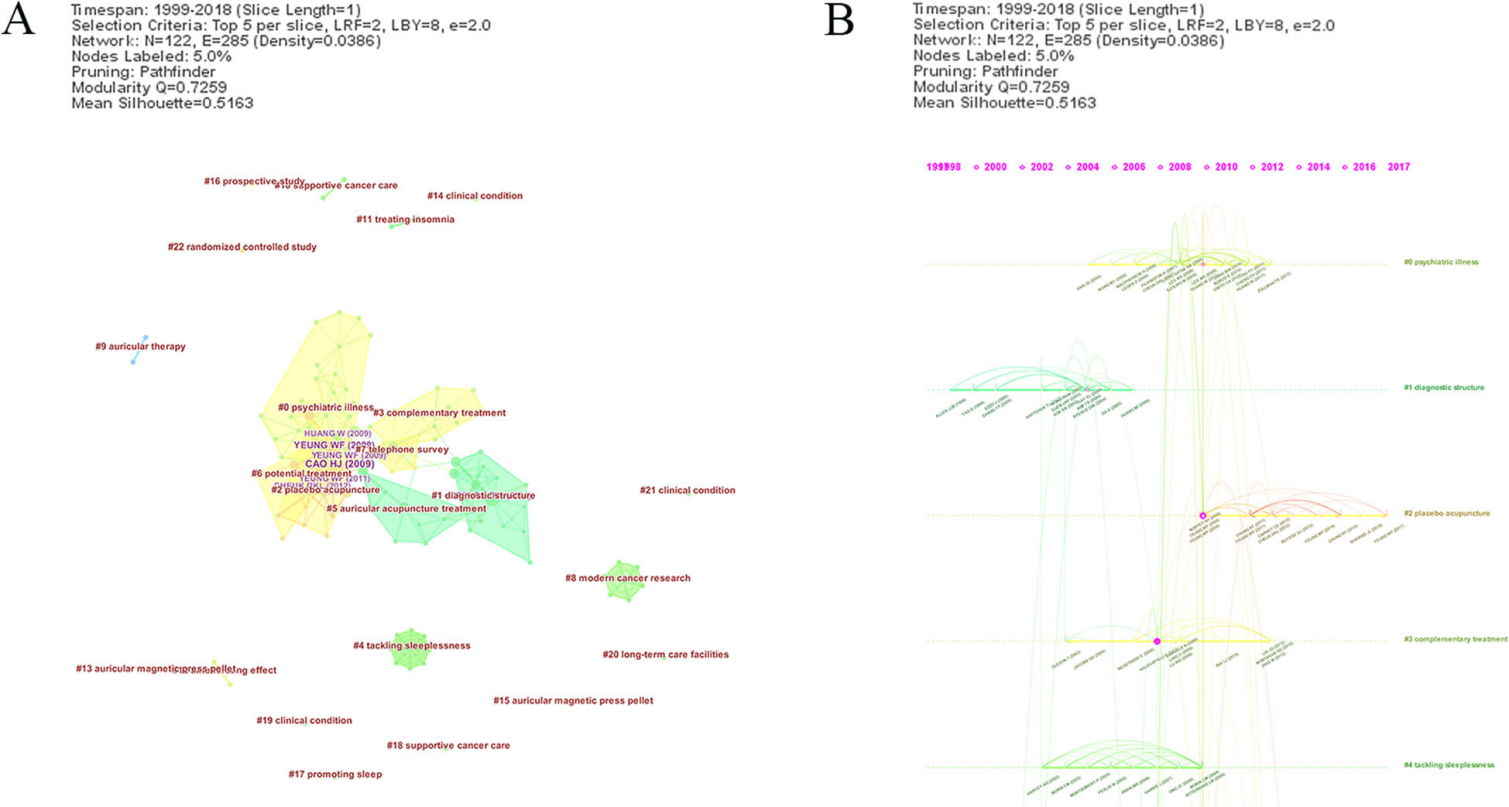
Cited reference map of acupuncture for insomnia. **a** The network map of cited reference related to acupuncture for insomnia. **b** The timeline view of cited reference related to acupuncture for insomnia

To get the key cluster of cited references, log-likelihood tests (LLR) was uesd to pick up the nounphrase from the title of the article in Citespace. Twenty-three clusters were generated to reflect the research patterns and emerging trends in network map (Fig. 4a). The largest cluster was “psychiatric illness” consisted of 26 references. The silhouette of this cluster was 0.8 which showed that the result was meaningfull. The most active citers to this cluster was a systematic review published by Huang W [40]. The second cluster was “diagnostic structure” with a silhouette of 0.893 and a member of 15. The value of the most active citer was 5.9519. “placebo acupuncture” and “complementary treament” were also active clusters. From the timeline view (Fig. 4b), Cluster 2 was painted in most warm color which meaned the lastest reaearch. There were related overlapping between Cluster 0 and Cluster 2, which indicated relevance in aspect of literature metrology.

### Analysis of keyword

Though analysising the frequncy and centality of keyword, research frontiers could be identified. From the network map of keyword consisted of 52 notes and 126 links, ‘randomized controlled trial’ had a high frequncy and centality. As the most common method to research the clinical efficiency, randomized controlled trials requied randomization, implementation of blinding in CONSORT (Consolidated Standards of Reporting Trials) statement [41]. For acupuncure therapy, it’s difficult to perform blindinng to patients and acupuncturist. In many trials, single blinding was used to perform blinding to assessors and subjects [30]. Electroacupuncture and acupressure are the common type of acupuncture therapy to treat insomnia. In primary insomnia and chronic insomnia, electroacupuncture had beneficial effect on sleep quality and the safety [31, 42]. As the different usage of electroacupuncture frequencies in studies, more attention should be paied to determine whether the electroacupuncture frequency is related to the treatment effect.

Insomnia is characterized by difficulty falling asleep, difficulty staying asleep (sleep maintenance disturbance), or poor quality (nonrestorative) sleep [43]. Mild insomnia can affect the function of daytime such as daytime fatigue, poor performance in work or school, decreased mood, resulting in reducing the quality of life [44]. More seriously, insomnia is not only a symptom of depression, but also a precursor of depression and is comorbid with major depression [45]. In the ranking list of centality (Table 6), ‘pain’ was in the front position to make us to investigate the relationship of insomnia and pain. In recent studies, insomnia is associated with chronic pain, and on the other hand insomnia can predict incidence of chronic pain [46].

**Table 6:** Top 10 keyword related to acupuncture for insomnia

Rank	Keyword	Freq	Rank	Keyword	Centality
1	acupuncture	133	1	acupuncture	0.91
2	insomnia	119	2	randomized controlled trial	0.45
3	randomized controlled trial	55	3	insomnia	0.29
4	sleep	28	4	sleep	0.24
5	depression	24	5	pain	0.14
6	electroacupuncture	21	6	acupressure	0.11
7	Metaanalysis	18	7	cognitive behavioral therapy	0.09
8	acupressure	14	8	auricular acupuncture	0.09
9	quality of life	10	9	electroacupuncture	0.08
10	cognitive behavioral therapy	8	10	metaanalysis	0.08

**Fig. 5:**
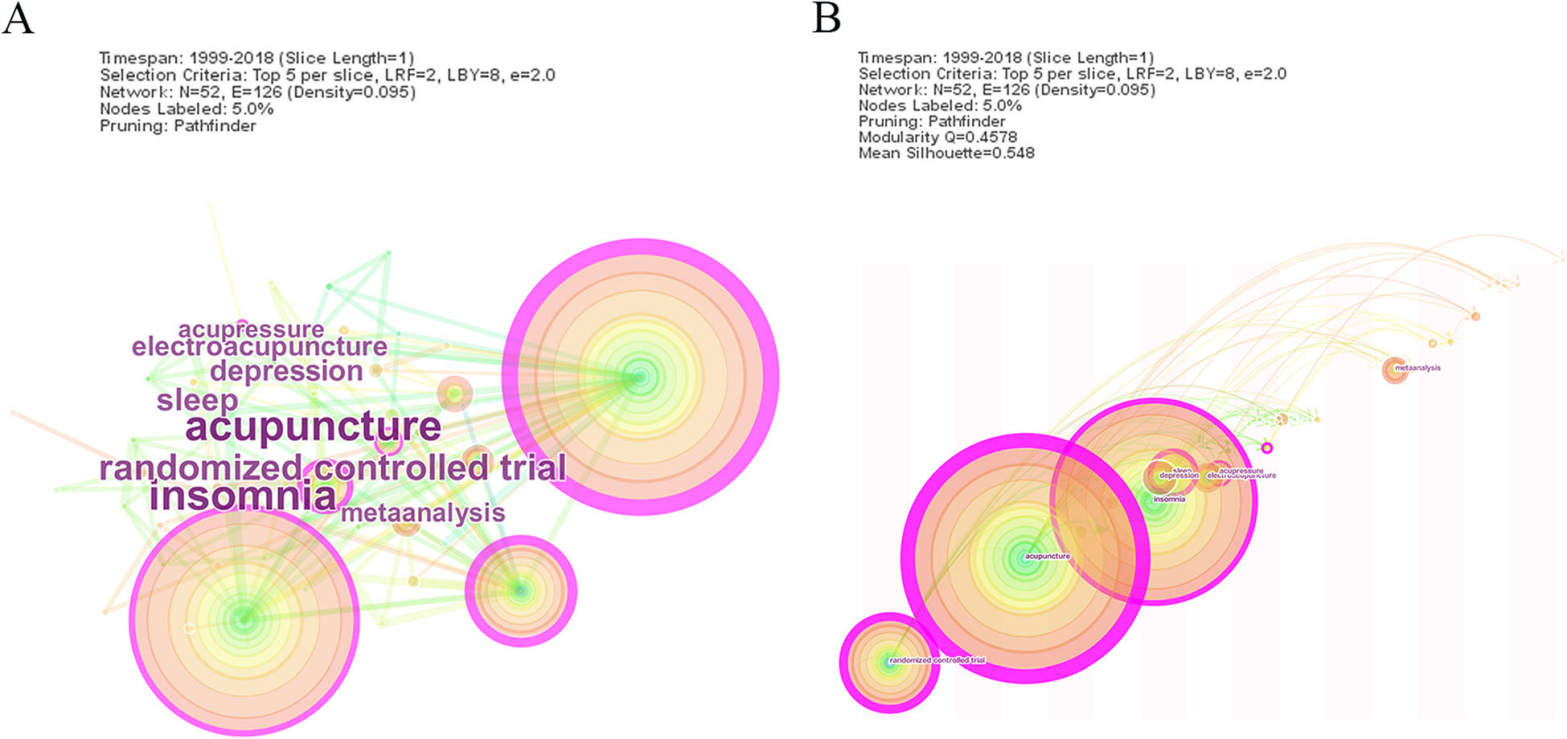
Keyword map of acupuncture for insomnia. **a** The network map of keyword related to acupuncture for insomnia. **b** The timezone view of keyword related to acupuncture for insomnia

Nowadays, systematic review is on the top of the level of evidence of therapeutic studies [47]. From the timezone view (Fig. 5), more and more attention was attracted on meta-ananlysis to evaluate the effectiveness of acupuncture therapy. Results showed that acupuncture therapy clould be benificial in clinical effective rate, sleep duration, sleep efficiency and adverse effect, but the quality of evidence was low [48]. It is suggested that studies should be more specific in details according to Reporting Interventions in Clinical Trials of Acupuncture (STRICTA).

## Conclusion

Using CiteSpace, bibliometric analysis of acupuncture therapy on insomnia from 1998 to 2018 were calculated. The rate of the annual publication gradually increased in the research trends. Insomnia is a sleep disorder related to the nervous system, a large number of references were published in the journals of sleep and neurology. As acupuncture therapy was part of alternative and complementry medicine, many articles relatred acupuncture therapy were cited in this field.

The top 5 of productive contries were China, USA, Taiwan, England and South Korea which widely distributed around the world. But more research was carried out in institutes of Hong Kong. Active authors also were mostly from Hong Kong. Obviously, a higher degree of acceptance acupuncture therapy was obtained in the Asian. In this article, we just analysised records from Web of Science (WoS) which most articles were in English. In the future, records in Chinese are not negligible to get more comprehensive study.

From the cited reference and keywords, systematic reviews and clinic trials were performed to confirm the effectiveness of acupuncture therapy. Randomized controlled studies mostly focused on electrocacupuncture and acupressure compared with sham acupuncture, medicine and placebo.

In conclusion, this study provids a perspective to the developing trend and hot topics of acupuncture therapy on insomnia. Citespace V is just a software to visualizing and analyzing network, we analyzed the research hotspots approximately. Deeper and more rigorous researches are needed in the future.

## Data Availability

The following information was supplied regarding data availability:The raw data can be directly obtained from the Web of Science Core Collection (WoSCC) of Thomson Reuters.

## Abbreviation

Freq: Frequency
